# Potential Utility of Cerebrospinal Fluid Glycoprotein Nonmetastatic Melanoma Protein B as a Neuroinflammatory Diagnostic Biomarker in Mild Cognitive Impairment and Alzheimer’s Disease

**DOI:** 10.3390/jcm12144689

**Published:** 2023-07-14

**Authors:** Julia Doroszkiewicz, Agnieszka Kulczyńska-Przybik, Maciej Dulewicz, Renata Borawska, Monika Zajkowska, Agnieszka Słowik, Barbara Mroczko

**Affiliations:** 1 Department of Neurodegeneration Diagnostics, Medical University of Bialystok, 15-269 Bialystok, Poland; agnieszka.kulczynska-przybik@umb.edu.pl (A.K.-P.); maciejdulewicz@gmail.com (M.D.); renata.borawska@umb.edu.pl (R.B.); monika.zajkowska@umb.edu.pl (M.Z.); mroczko@umb.edu.pl (B.M.); 2 Department of Neurology, Jagiellonian University, 30-688 Cracow, Poland; slowik@cm-uj.krakow.pl; 3 Department of Biochemical Diagnostics, Medical University of Bialystok, 15-269 Bialystok, Poland

**Keywords:** Alzheimer’s disease, GPNMB, YKL-40, neuroinflammation

## Abstract

Alzheimer’s disease (AD) is a very common neurodegenerative disorder characterized by the gradual loss of neurons and extracellular amyloid-peptide buildup. There is compelling evidence that the disease process depends on neuroinflammatory alterations, such as the activation of astrocytes and microglia cells. A transmembrane glycoprotein known as glycoprotein nonmetastatic melanoma protein B (GPNMB) plays a neuroprotective role during the development of neurodegeneration. To the best of our knowledge, this is the first investigation discussing the potential clinical usefulness of this protein in the AD continuum, especially in the MCI (mild cognitive impairment) stage. A total of 71 patients with AD or MCI as well as controls were enrolled in this study. The concentrations of GPNMB, YKL-40, Aβ1-42 (amyloid beta 1-42), Tau, and pTau and the Aβ1-42/1-40 ratio in the CSF (cerebrospinal fluid) were tested using immunological methods. The concentrations of both GPNMB and YKL-40 in the cerebrospinal fluid were significantly higher in patients with AD and MCI compared to the controls. Moreover, both proteins were biochemically associated with classical biomarkers of AD and were especially associated with the Aβ1-42/1-40 ratio and Tau and pTau levels in the whole study group. Elevated concentrations of GPNMB were observed in the Aβ(+) group of AD patients compared to the Aβ(−) subjects. Additionally, the diagnostic performance (AUC value) of GPNMB was higher than that of amyloid β1-42 in MCI patients compared with controls. Our study indicates that GPNMB might be a promising neuroinflammatory biomarker for the early diagnosis and prognosis of the AD continuum, with potential utility as a therapeutic target.

## 1. Introduction

Alzheimer’s disease (AD) is the most prevalent neurodegenerative disease and the most frequent cause of dementia [1]. It is a gradual illness, with up to 20 years before the first observable symptoms occur [2]. Memory loss, the inability to learn new things, aphasia, sleep cycle disruption, and serious issues with short- and long-term memory are some of its best-known symptoms [3]. The most prevalent characteristics of AD include the buildup of amyloid (Aβ) fibrils and insoluble plaques, neurofibrillary tangles (NFT) made of hyperphosphorylated Tau, neuronal and synaptic loss, and atrophy of brain regions important for memory [4]. A cascade of pathogenic events, including neuroinflammation and CNS (central nervous system) cell destruction, are triggered by the accumulation and aggregation of Aβ1-42 [5,6].

Despite the fact that neuroinflammation was described over 20 years ago, its role in the development of the disease is still unclear. Recent findings indicate that different Aβ complexes interact with expressed pattern recognition receptors in microglia and astrocytes, triggering innate immunity [7]. Depending on the disease stage, the innate immune response appears to have pleiotropic functions in the onset of cognitive impairment, with both neuroprotective and neurodegenerative outcomes. Activated microglia can be divided into two types: M1, being strictly connected to proinflammatory responses, and M2, described as neuroprotective [8]. In order to protect themselves, microglia change their phenotype. It has been described that, at the site of injury, M1 microglia typically predominate when the disease reaches its terminal stage and M2 microglia’s immune resolution and repair mechanisms are inhibited [8]. Without shielding healthy microglia, pathological brain processes can increase pro-inflammatory cytokine production and microglia hyperactivity. As a result, neurons are more susceptible to plaque formation and neurotoxic degradation, which enhances the pro-inflammatory response [9]. In AD, microglial activation is dynamic, and microglia constantly change between phenotypes. The dual role of microglia in AD pathogenesis led Fan et al. to hypothesize that there may be two peaks of microglial activation in AD: an early anti-inflammatory peak in the preclinical stage and a later pro-inflammatory peak in the clinical stage as illness develops after the failure in Aβ clearance [10,11]. A more recent theory suggests that a progressive change from a homeostatic to a disease-associated state is paralleled in microglia with the development of the illness. The downregulation of homeostatic genes and upregulation of known AD-associated genes, such as apolipoprotein E (APOE), triggering receptors expressed on myeloid cells 2 (TREM2), and TYRO protein tyrosine kinase-binding protein (TYROBP), are linked to the transition from normal microglia to disease-associated microglia (DAM) [12]. It should be noted that microglial activation occurs in two steps, the first of which is TREM2-independent, and the second of which is TREM2-dependent, which emphasizes the critical function of TREM2 in the development of neurodegenerative diseases. Transcriptomic research also supports the effects of aging on human microglial phenotypes, which include the upregulation of IL15, CXCR4, VEGF4 (encoding vascular endothelial growth factor 4), and RUNX3 and the downregulation of genes encoding cytoskeleton-associated proteins, cell surface receptors, and adhesion molecules [12,13].

The published data indicate that glycoprotein nonmetastatic melanoma protein B (GPNMB), an inflammatory molecule, is involved in the pathology underlying AD. GPNMB, also known as osteoactivin, is a type 1 transmembrane glycoprotein that was originally identified in low-metastatic melanoma cell lines in 1995 [14]. A significant amount of data also supports the idea that GPNMB functions as a negative regulator of inflammatory processes, which has interested neuroinflammatory researchers. Interestingly, GPNMB overexpression in macrophages decreased in vitro pro-inflammatory cytokine production [15]. Furthermore, it is suggested that GPNMB expression in the brain may be altered by pro-inflammatory stimuli [16]. LPS (lipopolysaccharide) is one of the factors responsible for neuroinflammation and is used as a model for this pathology, with a documented increase in proinflammatory cytokines after its administration in vitro and in vivo [17,18]. Rats that received LPS intraperitoneally (i.p.) had an increase in GPNMB-expressing cells, particularly in the postrema region. The overexpression of GPNMB correlated with OX42 expression, a marker for macrophages and microglia [19]. Furthermore, it was revealed that ADAM10, a metalloproteinase that cleaves APP and is involved in ectodomain shedding, can release GPNMB [20]. An increase in GPNMB levels in the brain has been found in a variety of neurodegenerative disorders, including AD [21], amyotrophic lateral sclerosis (ALS) [22], and Parkinson’s disease (PD) [23]. Nonetheless, the influence of GPNMB upregulation on the pathophysiology of these diseases has not yet been adequately explained. According to the available data, GPNMB appears to play a neuroprotective role. However, the mechanism is still not fully known. Recent publications showed that GPNMB promotes the polarization of macrophages to the “M2” type, which is described as more protective due to the release of anti-inflammatory cytokines such as IL-10 and TGF-β [21,24,25]. Therefore, GPNMB may possibly act as a factor influencing memory and synaptic plasticity, or working as a neuroprotective agent similar to carotenoids [26], flavonoids [27], coenzyme Q10 [28], or lutein [29]. 

YKL-40 is a chitin-binding lectin belonging to the glycosyl hydrolase family 18 which is also known as chitinase 3-like protein 1 (CHI3L1) or human cartilage glycoprotein 39 (HC-gp39) [30]. Numerous cell types, including macrophages, chondrocytes, neutrophils, and synovial fibroblasts, express the YKL-40 protein [31]. It was established that the higher production of YKL-40 by activated astrocytes and/or microglia is related to amyloid plaques and with NFT pathology [32]. In AD studies, they showed that, when compared to age-matched controls, the expression of mRNA for chitinase-3 like 3 (CHI3L3), a mouse homologue of YKL-40, was significantly higher in the brains of AD-model animals [33]. Remarkably, mRNA expression of TNF-α and YKL-40 was also significantly higher in brain samples obtained from patients with AD in comparison to controls [33]. 

Despite the mounting evidence of neuroinflammation occurring in AD, additional studies are necessary to clarify how these mechanisms are related to Aβ and tau pathologies and whether these connections are more significant in the beginning or in the later stages of the disease. Therefore, in the present study we compared the dynamic changes in pro- and anti-inflammatory proteins in different stages of AD. Despite the fact that both YKL-40 and GPNMB proteins are related to an inflammatory state in the CNS, they present opposite functions. While GPNMB is considered an anti-inflammatory protein, YKL-40 acts as proinflammatory protein. Combining these two proteins might provide interesting insight into the mechanisms of neuroinflammation in AD. There is still insufficient data regarding the possible diagnostic applicability of GPNMB in patients with mild cognitive impairments (MCI) and AD. To our best knowledge, there is only one publication assessing markers of microglial activity such as GPNMB in the CSF (cerebrospinal fluid) of AD patients and none describing the levels of this protein in the CSF of patients with MCI. Therefore, the present study aimed to assess the potential clinical significance of GPNMB in the AD continuum, especially in patients with MCI, in relation to other neuroinflammatory biomarkers (YKL-40) and classical biomarkers of AD that reflect amyloid and tau pathologies. 

## 2. Materials and Methods

### 2.1. Material

The study population consisted of 71 subjects (*n* = 50 women, *n* = 21 men; median age: 73 (51–89)) from the Department of Neurology, Jagiellonian University Hospital, Krakow, Poland, and included 35 AD patients (*n* = 28 women, *n* = 7 men; age: 75 (51–89)), 18 subjects with MCI (*n* = 11 women, *n* = 7 men; age: 75.5 (63–81)), and 18 non-demented controls (*n* = 11 women, *n* = 7 men; age: 67.5 (53–82)). Standard medical, physical, and neurological examinations, laboratory screening tests, a neurocognitive assessment, and magnetic resonance imaging or computed tomography of the brain were all employed in the clinical diagnosis of the study groups. Alzheimer’s disease cases with sporadic occurrences comprised the AD group. No patients involved in this study acknowledged a family history of Alzheimer’s disease in their medical interview. The National Institute on Aging and Alzheimer’s Association (NIA-AA) criteria were used to establish the AD diagnosis [34,35]. For the most accurate clinical diagnosis of AD, neurochemical data (levels of Aβ1-42, Tau, and pTau181 as well as values of the Aβ1-42/Aβ1-40 ratio) were combined with neuroimaging and neuropsychological tests. The MMSE score (range 0–30) was used to determine dementia severity (AD patients (MMSE: 22 (0–28)), MCI patients (MMSE: 27.5 (26–29)), and controls (MMSE: 28 (25–30)). The study was conducted in the Department of Neurodegeneration Diagnostics at the Medical University of Bialystok according to the guidelines of the Declaration of Helsinki; prior to any procedure, every patient signed an informed consent form, and the Bialystok University study (No. R-I-002/103/2019) received approval from the Ethics Committee. The exclusion criteria for the study included patients with suspected cerebrovascular disorders (such as cerebral hemorrhage, aortic aneurysm, intracranial aneurysm, stroke, or arteriovenous malformation), elevated albumin quotients (QAlb) indicative of blood–CSF barrier dysfunction, and changes in CT or MRI scans. 

The control group comprised individuals who were not experiencing subjective memory impairments and did not meet the MCI criteria but who might experience recurring headaches. None of the patients in this group displayed any meaningful changes in the levels of the recognized biomarkers for AD (Aβ1-42, Tau, and pTau181), which allowed the exclusion of the symptoms’ organic background. An Erlangen Score of 0 points across all 18 of the participants in this group supported these findings.

### 2.2. Biochemical Measurements

Lumbar punctures in the L4/L5 or L3/L4 interspace were used to collect CSF samples into polypropylene tubes. Prior to analysis, all CSF samples were centrifuged, aliquoted, and frozen at −80 °C. The concentrations of analyzed proteins (GPNMB, YKL-40, Aβ1-42, Aβ1-40, Tau, and pTau181) in the CSF were measured in the Department of Neurodegeneration Diagnostics, Medical University of Bialystok, Poland. 

For the assessment of concentrations of neurochemical dementia diagnostics (NDD) biomarkers, IBL kits (RE59661, RE59651, Hamburg, Germany) for Aβ1-42 and Aβ1-40 and Fujirebio kits (81572, 81574, Gent, Belgium) for Tau and pTau181 proteins were used.

The analysis of GPNMB was performed using Luminex Human Discovery assay plates, provided by R&D systems, Abingdon, UK, and a Luminex 200 analyzer (Thermo Fisher Scientific, Waltham, MA, USA) (multiplexing, multiparametric, fluorescence laser reading system on microspheres for the simultaneous determination of multiple parameters). Determination of YKL-40 was performed using an ELISA kit provided by MicroVue, Quidel, San Diego, CA, USA. According to the manufacturer’s protocols, duplicate measurements were assessed for each standard, control, and sample. 

### 2.3. Statistical Analysis

The PMCMRplus package in the statistical software RStudio (Version 1.4.1106, Boston, MA, USA) and Statistica 13.3 (StatSoft Polska, Krakow, Poland) were used to perform nonparametric tests. The Shapiro–Wilk test demonstrated that the protein concentrations were not distributed normally. The comparisons between the AD, MCI, and control groups were performed using the Kruskal–Wallis test. The post hoc Dwass–Steele–Critchlow–Fligner test was then used to assess significant differences between the levels of the tested groups in order to determine which groups had statistically significant differences. The results are presented as medians and interquartile ranges. Statistical significance was set at *p* < 0.05. In addition, the receiver operating characteristic (ROC) curve and area under curve (AUC) analysis was used to determine the diagnostic usefulness of the tested proteins as potential neuroinflammation-related biomarkers for AD.

## 3. Results

### 3.1. Patient Characteristics and CSF Concentrations of GPNMB and YKL-40

**Figure 1:**
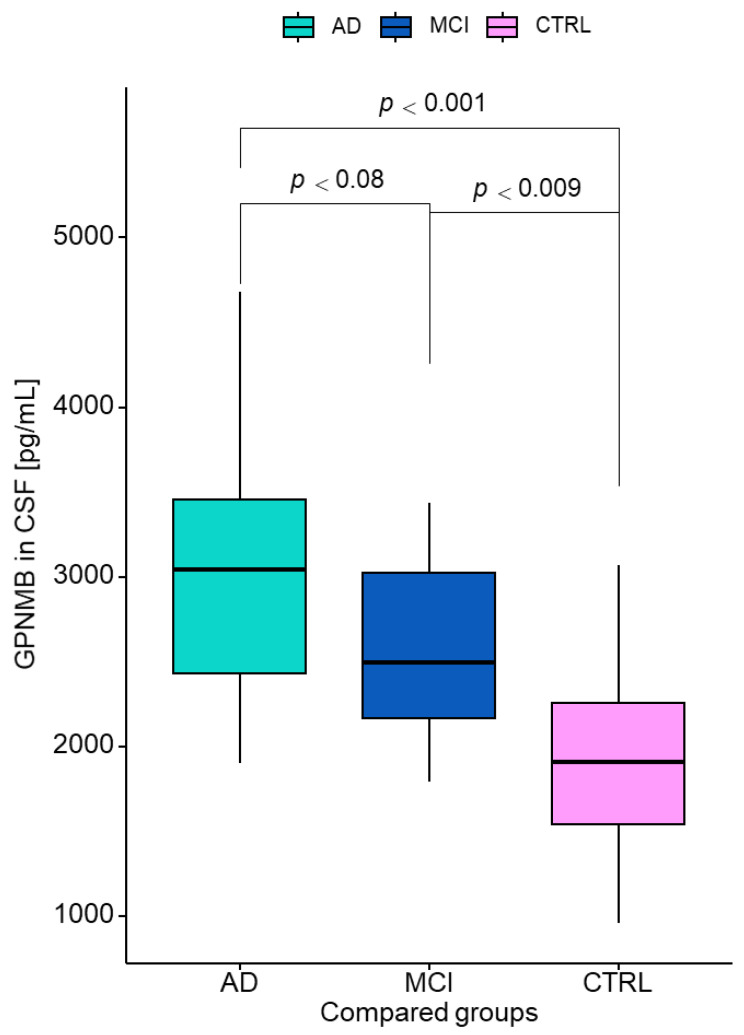
Cerebrospinal fluid levels of GPNMB by group. GPNMB—glycoprotein nonmetastatic melanoma protein B; AD—Alzheimer’s disease; MCI—mild cognitive impairment; CTRL—control; CSF—cerebrospinal fluid.

A summary of the CSF biomarker values in the examined groups are presented in Table 1. AD biomarkers were marked in all patients’ CSF samples. The Kruskal–Wallis test revealed statistically significant differences between all study groups for concentrations of GPNMB (*p* < 0.001, χ^2^ = 22) and YKL-40 in the CSF (*p* = 0.002, χ^2^ = 12.17), Aβ1-42 (*p* < 0.001, χ^2^ = 16.5), Tau (*p* < 0.001, χ^2^ = 47.1), and pTau181 (*p* < 0.001, χ^2^ = 41.7) as well as Aβ1-42/Aβ1-40 ratio (*p* < 0.001, χ^2^ = 33.7). The GPNMB levels in the CSF differed significantly between the patients with AD and the controls and between MCI patients and controls and are presented in Figure 1. These differences were verified by the post hoc Dwass–Steele–Critchlow–Fligner test. The highest CSF concentration of GPNMB was observed in the AD group in comparison to CTRL (*p* < 0.001) and MCI (*p* < 0.009) groups. In MCI patients, the CSF level of GPNMB was also higher than controls, but the difference was not statistically significant (*p* = 0.08). Additionally, we divided the AD and MCI groups into Aβ1-42(+) (AD = 17; MCI = 12) and Aβ1-42(−) (AD = 18; MCI = 6) subgroups according to a cut-off point of 538 pg/mL. The AD Aβ(+) group showed significantly higher concentrations of GPNMB than the AD Aβ(−) group (*p* = 0.028) (Table 2). The concentration of GPNMB in the MCI Aβ(+) was also higher than that of the MCI Aβ(−) group, although the difference was not significant.

**Figure 2:**
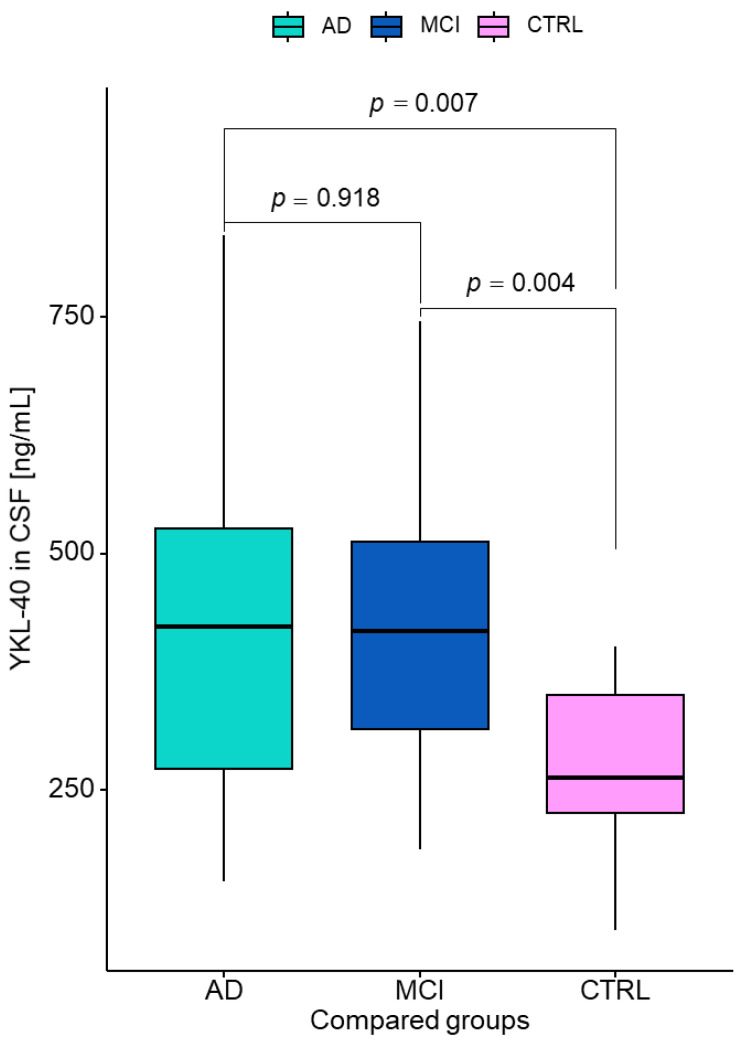
Cerebrospinal fluid levels of YKL-40 in AD, MCI, and CTRL groups. AD—Alzheimer’s disease; MCI—mild cognitive impairment; CTRL—control; CSF—cerebrospinal fluid.

The YKL-40 concentrations in the CSF also differed significantly between the patients with AD and the controls as well as between MCI patients and controls. A significantly higher concentration of YKL-40 was found in AD and MCI patients compared to controls (*p* = 0.007; *p* = 0.004). The concentrations in the MCI group were comparable with those of AD group (*p* = 0.918) (Figure 2).

### 3.2. Association between GPNMB, YKL-40, and CSF Biomarkers

**Figure 3:**
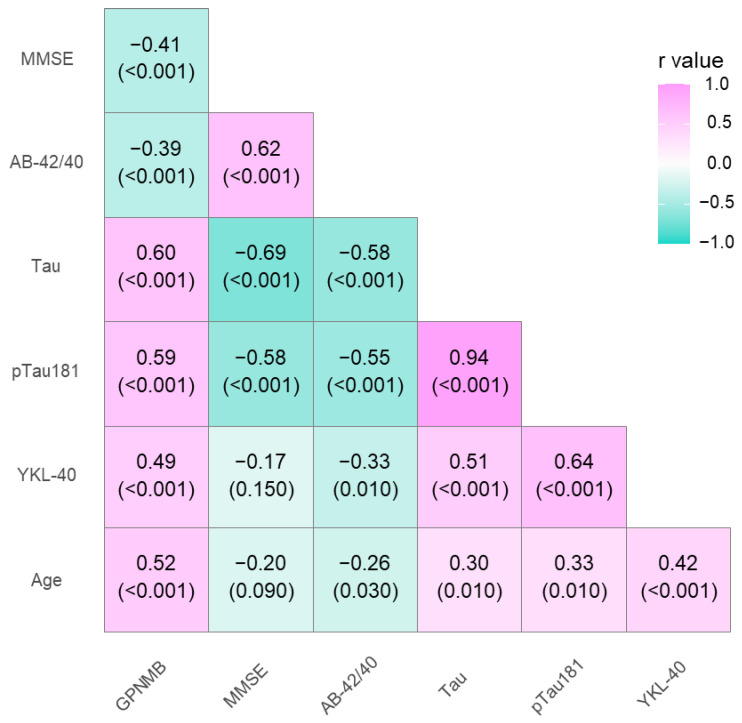
Spearman’s correlations between neurochemical biomarkers and tested proteins in the entire study population. MMSE—mini mental state examination score.

The associations between levels of GPNMB, YKL-40, and classical AD biomarkers were analyzed using the Spearman rank correlation test. In the entire study population, significant positive correlations were observed between the CSF levels of GPNMB and Tau (R = 0.6, *p* < 0.001), pTau (R = 0.59, *p* < 0.001), age (R = 0.52, *p* < 0.001), and YKL-40 in the CSF (R = 0.49, *p* < 0.001). A negative correlation was observed between GPNMB and the Aβ1-42/1-40 ratio (R = −0.39, *p* < 0.001) and MMSE (R = −0.41, *p* < 0.001). In addition, CSF YKL-40 levels in the entire population also correlated positively with Tau (R = 0.51, *p* < 0.001) and pTau (R = 0.64, *p* < 0.001) and negatively with the Aβ1-42/1-40 ratio (R = 0.33, *p* = 0.01) (Figure 3). 

In AD patients, the elevated CSF GPNMB level was significantly associated with YKL-40 (R = 0.5, *p* < 0.001) concentrations in the CSF and age (R = 0.62, *p* = 0.001). To determine the impact of the lower median age in the CTRL group, we selected age-matched groups of AD, MCI, and CTRL subjects (median age: 73, 74, and 73, respectively) and compared the concentrations of GPNMB in these groups. As a results of this analysis, we obtained similar statistical trends as were presented earlier (AD vs. CTRL *p* = 0.0029). A weaker correlation was observed between YKL-40 levels and Tau (R = 0.38, *p* = 0.02), but a stronger correlation was found between YKL-40 and pTau concentrations (R = 0.6, *p* > 0.001). 

In the MCI group, there were no significant correlations with GPNMB. However, CFS YKL-40 levels correlated positively with Tau (R = 0.78, *p* < 0.001), pTau (R = 0.86, *p* < 0.001), and Aβ1-42 (R = 0.56, *p* = 0.016) levels.

### 3.3. Diagnostic Usefulness of Candidate Biomarkers

**Figure 4:**
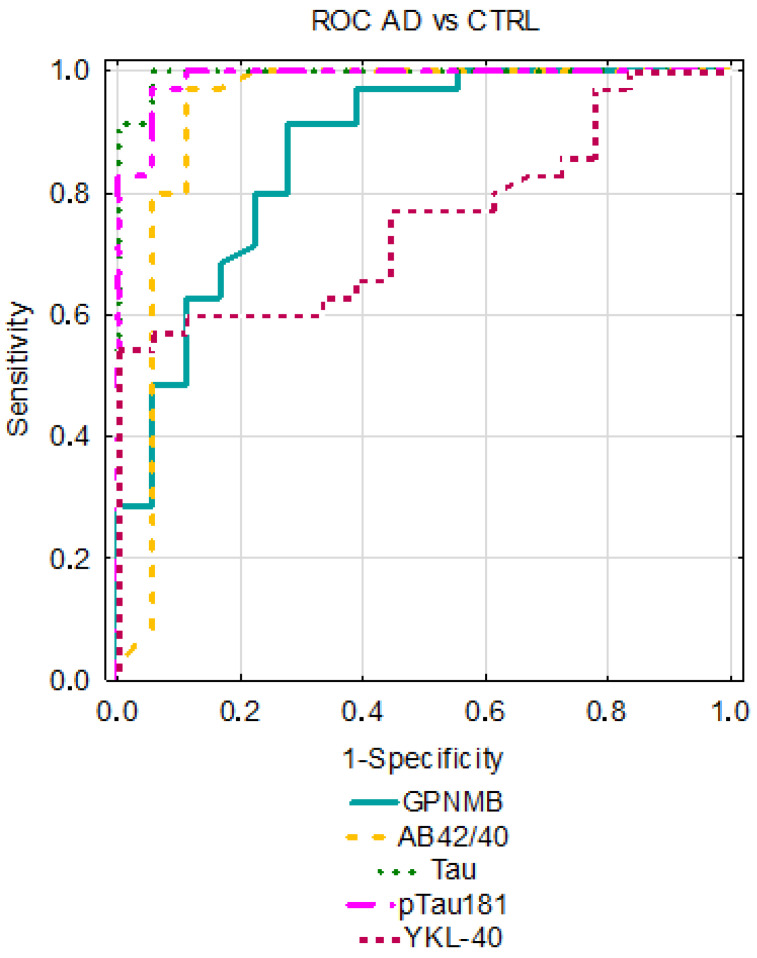
Comparison of area under ROC curves (AUC) for GPNMB, YKL-40, and classical biomarkers in AD between AD and CTRL groups.

**Figure 5:**
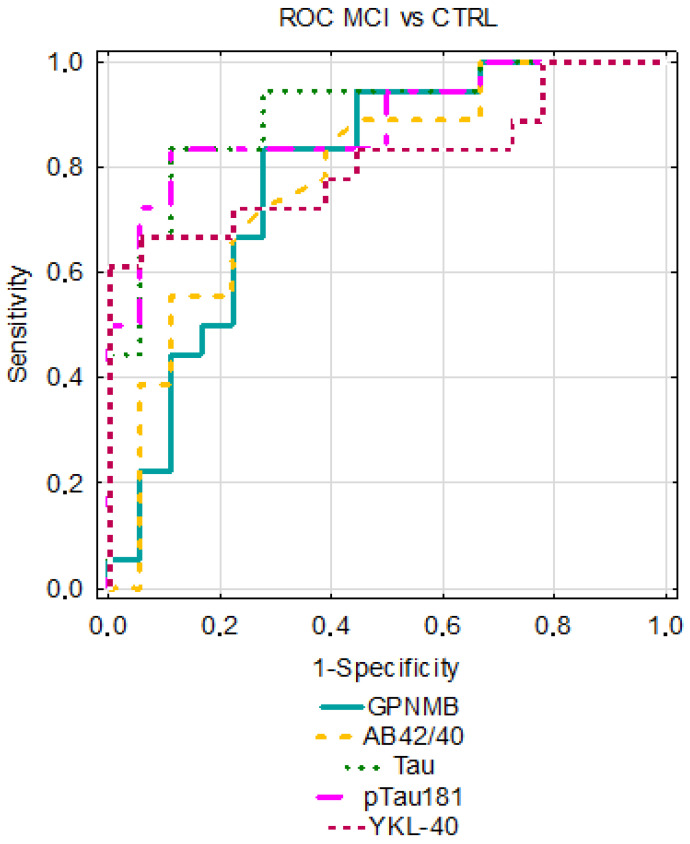
Comparison of area under ROC curves (AUC) for GPNMB, YKL-40, and classical biomarkers between MCI and CTRL groups.

The analysis of the receiver operating characteristic curves (ROCs) was performed for the AD and MCI groups compared to the CTRL group. The AUC for GPNMB was better than that assessing Aβ1-42 alone; however, it was slightly lower in comparison to the Aβ1-42/1-40 ratio and Tau proteins in differentiating between the AD and CTRL groups. In AD, the lowest diagnostic usefulness was presented with the YKL-40 concentrations. Similar results for GPNMB and YKL-40 were observed in MCI patients vs. controls; their AUC values were higher than that of Aβ1-42 and the Aβ1-42/1-40 ratio and lower than that of the Tau proteins. The results are presented in Table 3 and Figure 4 and Figure 5.

## 4. Discussion

The course and severity of the disease in AD are influenced by the ongoing activation of microglia and astrocytes in response to misfolded and aggregated proteins causing inflammation [36]. Thus, studying the proteins that represent neuroinflammation as markers of illness progression and the emergence of cognitive impairments seems crucial, especially when we know that targeting amyloid plaques and neurofibrillary tangles as a therapeutic approach is not effective. Taking this information into account, we proposed a potential novel biomarker related to neuroinflammation (GPNMB) to facilitate and improve the early diagnosis of AD. In the APP/PS1KI and 5XFAD mouse models of AD, GPNMB was shown to be significantly elevated in a subpopulation of microglia cells [21,37]. Moreover, another study revealed that GPNMB is able to improve memory in mice, which supports the neuroprotective function of this protein [38].

In our study, we assessed the GPNMB levels in the CSF of AD patients and a control group (i.e., individuals without cognitive decline). The highest GPNMB concentrations were observed in the CSF of AD patients compared to MCI subjects and older people without cognitive decline. Moreover, our findings demonstrated that CSF GPNMB concentrations had already increased in the early clinical stages of cognitive decline (MCI) and continued to rise with disease severity. This may indicate the possible application of this protein as a disease severity biomarker. Our results support the two main hypotheses describing the role of activated microglia in brain disorders. It is suggested that the increased levels of microglia-activated proteins, such as GPNMB, reflect the activation of protective immunological mechanisms in the brain, which leads to an increased clearance of accumulated pathological proteins by phagocytosis. This phenomenon seems to be crucial for microglia to defend the CNS from damage such as the accumulation of β-amyloid. On the other hand, a number of studies have shown that persistent, progressive microglial activation is detrimental to neurons and, with time, increases the severity and course of the disease. In AD, protective microglial activities are thought to occur in the early stages of the illness despite the fact that the negative effects of microglia activation tend to manifest in later stages of the disease [39]. Our findings are similar to previous studies, where the CSF levels of GPNMB in AD patients were higher than in controls [21]; however, there are conflicting results in the literature [40]. The study by Aichholzer et al. showed no significant increase in the concentrations of this protein, although the authors pointed out limitations that included a lack of information about the inflammatory state of the subjects from the control group [40]. Moreover, the studies by Bai et al. and Wang et al. detected the presence of GPNMB in the cortex, CSF, and serum of AD patients using a multimodal proteomic method, with the elevated CSF levels confirmed by an alternative method (ELISA) [41,42]. The findings from mouse models and human brains demonstrate that GPNMB is largely concentrated in microglial cells around extracellular Aβ deposits in the brain tissues of patients [21,43]. Moreover, the findings of our study showed higher concentrations of GPNMB in AD Aβ(+) patients in comparison to the Aβ(−) group, indicating a connection to amyloid pathology, which may be a result of the neuroprotective activity of GPNMB in the brain areas affected by amyloid plaques. Similar observations were described in Aichholzer et al.’s study [40], in which the AD group was also divided into Aβ-positive and Aβ-negative subgroups through PET imaging, showing higher concentrations of GPNMB in the positive group but without statistical significance [40]. However, GPNMB is not a specific biomarker for AD; overexpression of this protein was also observed in other neurodegenerative diseases, such as ALS and PD. In an ALS study, targeted multiple reaction monitoring mass spectrometry was used to quantify CSF proteins, and individuals with short-lived ALS had higher GPNMB levels [44]. This was confirmed by a newer, autonomous cohort [45]. Furthermore, overexpression and elevated concentrations of GPNMB were observed in a 1-methyl-4-phenyl-1,2,3,6-tetrahydropridine mouse model of PD [46]. Interestingly, examination of recently frozen post-mortem human brain samples from patients with sporadic Parkinson’s disease revealed higher GPNMB levels in the substantia nigra of PD patients in comparison to healthy control participants [23]. 

We also compared the GPNMB levels with another well-known proinflammatory protein—YKL-40. Our study showed significantly higher levels of this protein in the CSF of AD patients compared to MCI subjects and the control group. This is consistent with previous publications and meta-analyses as well as with described trends [47,48]. Moreover, these revelations can confirm the microglia polarization taking place in the early and fully developed disease stages. Furthermore, YKL-40 concentrations correlated with Tau, suggesting an association between tauopathies and AD, which supports earlier research [49]. According to these findings, GPNMB and YKL-40 may help distinguish early stages of dementia (MCI) from cognitively normal subjects, as well as AD dementia from controls without cognitive impairments. Despite both proteins being elevated in the CSF of the cognitively impaired patients, they probably reflect different functions regarding neuroinflammation. YKL-40 is described as a proinflammatory protein released in microglia activation, while GPNMB has anti-inflammatory abilities. A study by Hüttenrauch et al. [21] revealed that GPNMB inhibits the inflammatory response during neurodegenerative processes, which might be protective for neurons by attenuating neuroinflammation. Changes in levels of molecules related to the inflammatory state in the brain demonstrate how the microglia phenotype is constantly changing during the development of the disease. These changes in inflammatory proteins might suggest potential diagnostic utility in using these proteins to monitor disease progression [21]. 

GPNMB levels correlated positively with Tau proteins in the entire study population, similar to YKL-40. Remarkably, it was demonstrated that Tau can also induce microglia activation in the course of AD progression [50]. Thus, this relationship between GPNMB, YKL-40, and Tau proteins can be interpreted as the influence of neuroinflammation on accelerated neurodegeneration, which is reflected by the progressive cognitive decline in patients. In addition, in our study, YKL-40 also correlated with the Aβ1-42/1-40 ratio in all groups and in the AD group. Amyloid metabolism disturbances have a positive influence on ongoing inflammatory processes in the diseased brain; therefore, we observed higher levels of inflammatory proteins. 

Moreover, we assessed the diagnostic usefulness of the tested proteins based on AUC results. To our knowledge, this paper is the first to assess these parameters regarding GPNMB levels in MCI. GPNMB levels showed better discriminatory capability than Aβ1-42 levels and the Aβ1-42/1-40 ratio in differentiating between the MCI and CTRL groups. Similarly, the AUC value for GPNMB was higher than that of Aβ1-42 but lower than other classical biomarkers (Aβ1-42/1-40 ratio, Tau, pTau) in the AD group compared to the CTRL group. The AUC for YKL-40 was slightly higher than that of GPNMB in MCI patients compared to controls but slightly lower in the AD vs. CTRL group. Our results indicate a comparable clinical utility of both proteins. 

Our study has several limitations; therefore, it is important to interpret the results carefully. First, despite the fact that our study focused on a rather small population, we carefully selected the group of patients with cognitive decline (MCI and AD) based on biomarker biochemistry. Moreover, these patients had been diagnosed in the same center and treated within similar time periods. To further prevent the impact of various conditions on the evaluated biomarker levels in our study population, the collection techniques were also standardized. 

## 5. Conclusions

The results from our study suggest a potential clinical application for the GPNMB protective factor as a biomarker of the Alzheimer’s disease continuum. We observed a relationship between the altered amyloid metabolism and concentrations of GPNMB, which indicates a role for this protein in the pathogenesis of the disease. Moreover, elevated levels of GPNMB in the Aβ(+) group indicate the potential diagnostic usefulness of this protein. In the early stages of cognitive impairment (MCI group), GPNMB demonstrated better diagnostic performance (AUC value) than amyloid β proteins and was comparable to the Aβ1-42/1-40 ratio. Additionally, the CSF levels of this protein increased with the severity of the disease, suggesting the possible application of this biomarker in monitoring disease progression. 

## Figures and Tables

**Table 1:** The concentrations of tested proteins in the study groups.

Tested Variables	Median (Interquartile Range)			*p* (Kruskal–Wallis Test)	*p* (Dwass–Steele–Critchlow–Flinger Test)		
	AD	MCI	Controls		AD vs. CTRL	AD vs. MCI	MCI vs. CTRL
Aβ1-42 (pg/mL)	520 (197–994)	802 (382–1878)	851 (357–1235)	<0.001	<0.001	0.030	0.947
Aβ1-42/1-40 ratio	0.03 (0.02–0.05)	0.04 (0.03–0.07)	0.07 (0.02–0.09)	<0.001	<0.001	<0.001	0.011
Tau (pg/mL)	710 (357–1722)	389 (209–994)	221 (148–414)	<0.001	<0.001	<0.001	<0.001
pTau181 (pg/mL)	87.9 (46.4–209)	57.2 (34.1–97.1)	36.6 (24.8–55.6)	<0.001	0.001	<0.001	0.002

**Table 2:** The concentrations of GPNMB in AD and MCI patients based on amyloid Aβ status (* *p* < 0.05). GPNMB—glycoprotein nonmetastatic melanoma protein B; AD—Alzheimer’s disease MCI—mild cognitive impairment.

	*n*	GPNMB Median	Interquartile Range	*p*-Value
AD	Aβ(+) = 17	3328	2830–3842	0.028 *
	Aβ(−) = 18	2559	2276–3397	
MCI	Aβ(+) = 12	2658	2366–3328	0.122
	Aβ(−) = 6	2174	1923–2950	

**Table 3:** AUC of tested parameters in compared groups.

Tested Parameters	ROC Criteria in AD Compared to CTRL				ROC Criteria in MCI Compared to CTRL			
	AUC	SE	95% C.I. (AUC)	*p* (AUC = 0.5)	AUC	SE	95% C.I. (AUC)	*p* (AUC = 0.5)
GPNMB	0.869	0.054	0.763–0.975	<0.001	0.787	0.078	0.633–0.941	0.003
YKL-40	0.755	0.065	0.627–0.882	0.001	0.812	0.075	0.665–0.959	<0.001
Aβ1-42	0.836	0.063	0.713–0.958	<0.001	0.531	0.102	0.33–0.731	0.763
Aβ1-42/1-40 ratio	0.934	0.052	0.833–0.964	<0.001	0.784	0.078	0.631–0.937	0.003
Tau	0.995	0.006	0.984–1	<0.001	0.901	0.052	0.8–1	<0.001
pTau181	0.989	0.011	0.967–1	<0.001	0.883	0.057	0.77–0.995	<0.001

## Data Availability

The data presented in this study are available on request from the corresponding author.
